# The Effects of Exogenous 2,4-Epibrassinolide on the Germination of Cucumber Seeds under NaHCO_3_ Stress

**DOI:** 10.3390/plants13030394

**Published:** 2024-01-29

**Authors:** Wenjing Nie, Biao Gong, Bing Geng, Dan Wen, Peng Qiao, Hongen Guo, Qinghua Shi

**Affiliations:** 1 Yantai Engineering Research Center for Plant Stem Cell Targeted Breeding, Shandong Institute of Sericulture, Yantai 264001, China; cottage1990@163.com (W.N.);; 2 Stage Key Laboratory of Crop Biology, College of Horticulture Science and Engineering, Shandong Agricultural University, Tai’an 271018, China

**Keywords:** cucumber, salt-alkaline stress, brassinolide, alleviating effect, seed germination

## Abstract

This investigation focused on the suppressive impact of varying NaHCO_3_ concentrations on cucumber seed germination and the ameliorative effects of 2,4-Epibrassinolide (EBR). The findings revealed a negative correlation between NaHCO_3_ concentration and cucumber seed germination, with increased NaHCO_3_ concentrations leading to a notable decline in germination. Crucially, the application of exogenous EBR significantly counteracted this inhibition, effectively enhancing germination rates and seed vigor. Exogenous EBR was observed to substantially elevate the activities of superoxide dismutase (SOD), catalase (CAT), and peroxidase (POD), thereby mitigating oxidative damage triggered under NaHCO_3_ stress conditions. Additionally, EBR improved enzyme activity under alkaline stress conditions and reduced starch content in the seeds. Pertinently, EBR upregulated genes that were associated with gibberellin (GA) synthesis (*GA20ox* and *GA3ox*), and downregulated genes that were linked to abscisic acid (ABA) synthesis (*NCED1* and *NCED2*). This led to an elevation in GA3 concentration and a reduction in ABA concentration within the cucumber seeds. Therefore, this study elucidates that alleviating oxidative stress, promoting starch catabolism, and regulating the GA and ABA balance are key mechanisms through which exogenous EBR mitigates the suppression of cucumber seed germination resulting from alkaline stress.

## 1. Introduction

Saline-alkali stress profoundly affects essential plant processes, including germination, growth, and photosynthesis, thereby posing challenges to sustainable horticultural practices [1,2]. This stress can be classified into two distinct classifications: salt stress, attributable to neutral salts, and alkali stress, caused by alkaline salts [3]. Both types instigate ionic toxicity and osmotic imbalances, culminating in oxidative stress. Of particular note, the heightened pH associated with alkali stress renders it more injurious to plants than salt stress [1,4,5]. Such stress promotes reactive oxygen species (ROS) accumulation, engendering oxidative harm. This not only disrupts the internal hormonal balance but also diminishes enzymatic activity, further suppressing plant growth [1,6,7]. While extensive research has elucidated mechanisms underpinning plant salt tolerance, our understanding of plant adaptations to alkali stress remains comparatively nascent [5].

During the initiation of germination, the seed’s embryo transitions from a quiescent state to heightened physiological activity. This metamorphosis begins with suckering and culminates as the radicle penetrates the seed coat, prompting the elongation of the radicle and the embryonic axis [8]. Seed germination, the pivotal onset in the life cycle of a plant, plays a determinative role in influencing subsequent phases of growth, maturation, and overall plant development [9,10]. Given the inherent immobility of plants, the strategic oscillation between dormancy and germination becomes imperative for their evolutionary continuity and reproductive process [10,11,12]. In an agronomic context, the precision and robustness of seed germination bear directly upon the quality of the seedlings and the eventual crop yield. The germination process can be segmented into suckering, activation, and emergence [9,11,13]. Following water uptake, the seeds undergo volumetric expansion, triggering a series of metabolic enzyme activations. This process is accompanied by significant changes in the internal hormonal balance, notably a surge in gibberellin (GA) synthesis and a marked reduction in abscisic acid (ABA) levels. These molecular alterations effectively terminate dormancy, enabling the embryonic axis and roots to penetrate their surrounding tissues, culminating in the completion of germination [11,13,14,15]. This complex orchestration involves an array of physiological and biochemical operations, including the catabolism of organic substrates, the genesis and modification of RNA and proteins, hormone synthesis, and overarching metabolic realignments [11,14,16].

Brassinosteroids (BRs), often designated as “the sixth plant hormone”, are a collection of approximately 40 steroid hormones that are ubiquitous in plants [17]. They have a crucial function in enhancing a plant’s resilience against an extensive array of both biotic and abiotic stress factors, encompassing various environmental and biological challenges. Central to numerous physiological processes, BRs are integral for seed germination, cell elongation, cotyledon expansion, dark morphogenesis, and ethylene production initiation [17,18]. Plant adaptability to various biotic and abiotic stressors is a critical area of horticultural research. The germination phase is especially vulnerable to environmental stressors such as salinity, drought, heavy metals, and extreme temperatures. Accumulating evidence has highlighted the instrumental role of BRs in counteracting these challenges [12]. Specifically, under saline conditions, BRs have been empirically demonstrated to ameliorate membrane damage, reduce seed deterioration, and enhance the intrinsic salt tolerance in non-halophytic plants [19,20]. Brassinosteroids (BRs) play a crucial part in the process of seed germination. While they are essential for standard germination, the Arabidopsis mutants *det2-1* (involved in BR biosynthesis) and *bri1-1* (BR-insensitive) show increased susceptibility to ABA-mediated inhibition compared to the wild-type counterparts. Notably, exogenous BRs facilitate germination in both gibberellin (GA) synthesis mutants and GA-insensitive mutants [12].

As a globally cultivated crop, cucumber is notably susceptible to salinity stress, whether grown in open fields or greenhouses [1,21]. This susceptibility intensifies when coupled with soil crusting, elevating the risk of pathogenic attacks on seeds and seedlings [5,22,23]. Alkaline stress, distinguished by its high pH, poses a distinct challenge, exerting more pronounced inhibitory effects on plant growth and germination than neutral salts [24,25,26]. Our research examines the role of externally applied 2,4-Epibrassinolide (EBR) in counteracting the detrimental effects of alkalinity on cucumber seed germination, shedding light on its remedial properties and the integral physiological processes involved. Our research seeks to establish a foundational understanding that supports the strategic application of EBR in confronting alkaline stress challenges in cucumber cultivation.

## 2. Results

**Figure 1:**
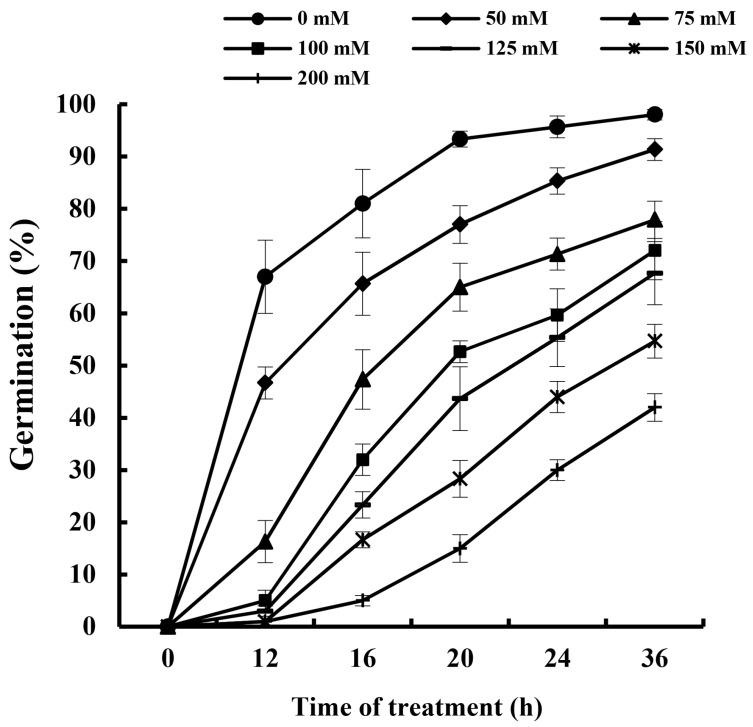
Effects of NaHCO_3_ with different concentrations on germination rate of cucumber seeds. Treatments include (1) Control (CK) with distilled water, representing the baseline condition; (2) Stress (S), induced by exposure to 75 mmol L^−1^ NaHCO_3_, simulating alkaline stress; and (3) Stress Mitigation with EBR (S + EBR), a combined treatment of 0.2 μmol L^−1^ EBR and 75 mmol L^−1^ NaHCO_3_, demonstrating EBR’s efficacy in stress response modulation. Seeds were pretreated for 12 h in either distilled water or a 0.2 μmol L^−1^ EBR solution prior to the commencement of germination tests.

The germination rate is an essential measure of seed vigor and is vital in the germination process. Typically, cucumber seeds exhibit a 95% germination rate within 24 h. However, the addition of external NaHCO_3_ notably suppressed this rate, with the inhibition intensifying as NaHCO_3_ concentrations increased. Figure 1 illustrates these effects. At a concentration of 200 mmol L^−1^ NaHCO_3_, the germination rate drastically dropped to 30% after 24 h and 42% after 36 h. For the purpose of this study, a NaHCO_3_ concentration of 75 mmol L^−1^ was selected, at which the germination rate was approximately 30% at 24 h and 42% at 36 h.

**Figure 2:**
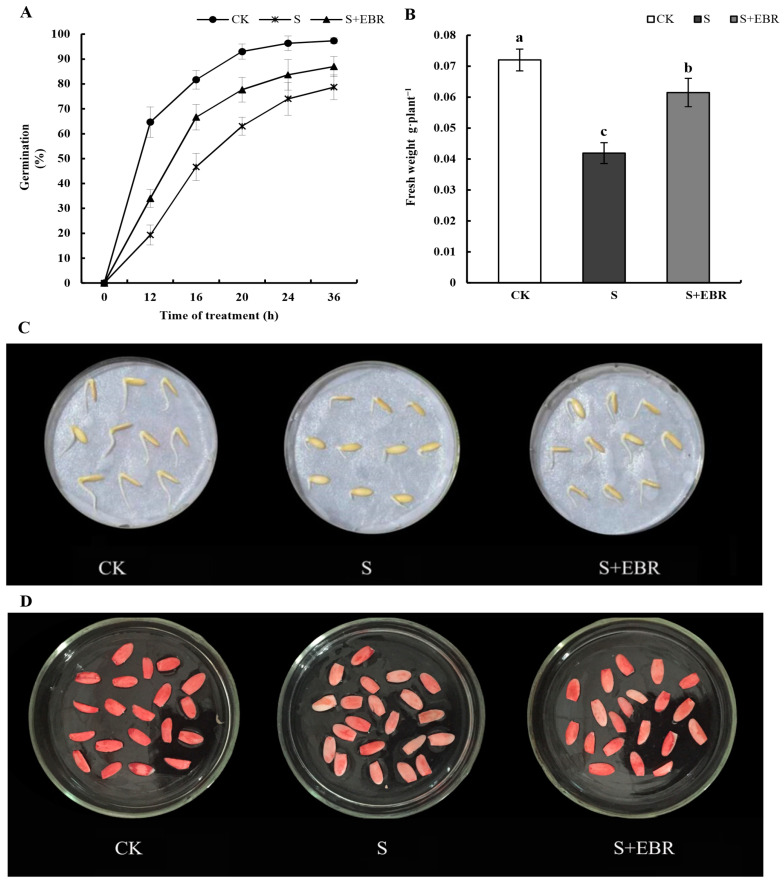
Effects of exogenous EBR on germination rate (**A**), fresh weight (**B**), phenotypes (**C**), and vigor (**D**) of cucumber seeds under NaHCO_3_ stress. Treatments include (1) Control (CK) with distilled water, representing the baseline condition; (2) Stress (S), induced by exposure to 75 mmol L^−1^ NaHCO_3_, simulating alkaline stress; and (3) Stress Mitigation with EBR (S + EBR), a combined treatment of 0.2 μmol L^−1^ EBR and 75 mmol L^−1^ NaHCO_3_, demonstrating EBR’s efficacy in stress response modulation. Seeds were pretreated for 12 h in either distilled water or a 0.2 μmol L^−1^ EBR solution prior to the commencement of germination tests. Different letters above bars indicate significance among different treatments as determined using Duncan’s samples comparison test (*p* < 0.05).

Figure 2 underscores the significant effect of exogenous EBR on the germination rate and percentage of cucumber seedlings under NaHCO_3_ stress (*p* < 0.05). After 20 h of treatment, the germination rate was recorded at 63%, which increased to 77.67% following the addition of EBR. After 36 h, EBR application led to a notable 46.67% increase in the fresh weight of stressed seeds (*p* < 0.05), significantly enhancing radicle elongation. TTC staining indicated limited and pale staining in seeds under NaHCO_3_ stress, while the inclusion of EBR resulted in a marked increase in stained seeds with deeper coloration.

**Figure 3:**
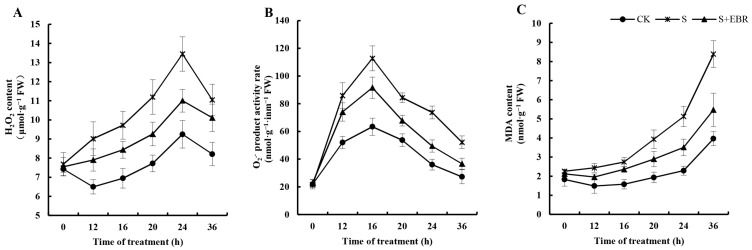
Effects of EBR on H_2_O_2_ content (**A**), O_2_^−^ product activity rate (**B**), and MDA content (**C**) in the process of cucumber seed germination under NaHCO_3_ stress.

Figure 3 distinctly shows that NaHCO_3_ stress induced a significant rise in the levels of H_2_O_2_, O_2_^−^ generation rate, and MDA content in cucumber seeds, illustrating a marked increase in oxidative stress compared to the control group (*p* < 0.05). Importantly, the introduction of exogenous EBR markedly mitigated these stress indicators, demonstrating a significant reduction in the concentrations of ROS and malondialdehyde (MDA) in seeds exposed to NaHCO_3_ (*p* < 0.05). This highlights the pivotal role of EBR in reducing the oxidative harm resulting from alkaline stress.

**Figure 4:**
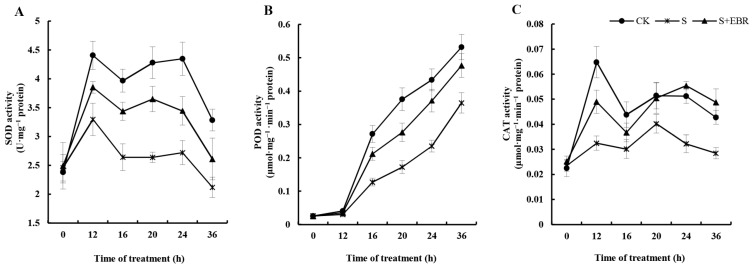
Effects of exogenous EBR on SOD (**A**), POD (**B**), and CAT (**C**) activity in the process of cucumber seed germination under NaHCO_3_ stress.

Moreover, the application of NaHCO_3_ during germination notably diminished the activities of crucial antioxidant enzymes, including superoxide dismutase (SOD), peroxidase (POD), and catalase (CAT), when contrasted with the control group (Figure 4). The addition of exogenous EBR, however, not only counteracted this decline but also notably enhanced the activities of these enzymes under NaHCO_3_ conditions. These observations indicate exogenous EBR strengthens the antioxidative defense mechanism in cucumber seeds, promoting ROS scavenging during germination in alkaline conditions.

**Figure 5:**
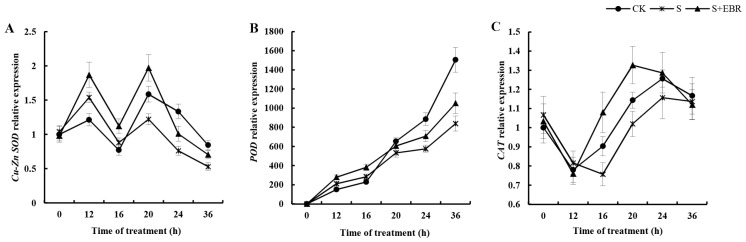
Effects of exogenous EBR on gene expression of *Cu-Zn SOD* (**A**), *POD* (**B**), and *CAT* (**C**) in the process of cucumber seed germination under NaHCO_3_ stress.

Figure 5 reveals crucial observations regarding the regulatory impact of exogenous EBR upon antioxidative gene activity within cucumber seeds exposed to NaHCO_3_. Exogenous EBR significantly increased the activity of genes such as *Cu-Zn SOD*, *CAT*, and *POD* under NaHCO_3_ stress conditions. This upregulation was consistently observed for *POD* and *Cu-Zn SOD* genes throughout the experimental period. In particular, the elevation in the expression of the *CAT* gene was predominantly significant between 12 and 24 h after initiating NaHCO_3_ treatment. These findings highlight the specific role of EBR in upregulating crucial antioxidative genes, thereby potentially mitigating the oxidative stress effects induced by NaHCO_3_ in cucumber seed germination.

**Figure 6:**
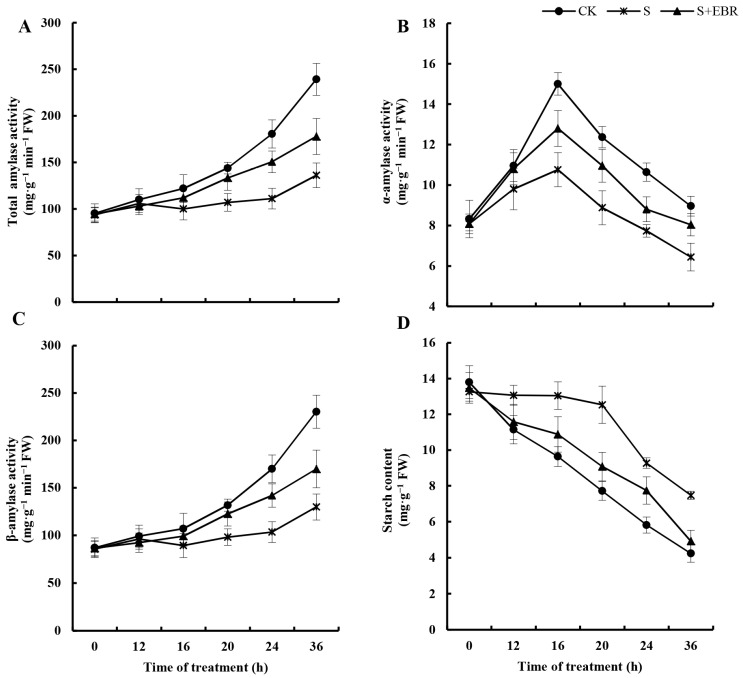
Effects of EBR on activities of total amylase (**A**), α-amylase (**B**), β-amylase activity (**C**), and the starch content (**D**) in the process of cucumber seed germination under NaHCO_3_ stress.

Figure 6 offers a comprehensive profile of amylase activity in cucumber seeds during germination. Initially, there was a spike in α-amylase activity, which then decreased, juxtaposing the consistent rise in total activities of amylase and β-amylase. Alongside this, a steady decline in the content of starch was observed. Under the NaHCO_3_ condition, amylase activity was significantly reduced, while starch content increased compared to the control. The introduction of exogenous EBR, however, led to an elevation in α-amylase activity and a reduction in starch content under alkali stress. In the initial 12 h period of NaHCO_3_ treatment, the influence of EBR on β-amylase and total amylase activities was minimal. After this period, EBR substantially augmented both activities. These observations suggest that EBR effectively counters the challenges to cucumber seed germination induced by alkali stress, primarily via increasing the activity of amylase and aiding in the breakdown of starch.

**Figure 7:**
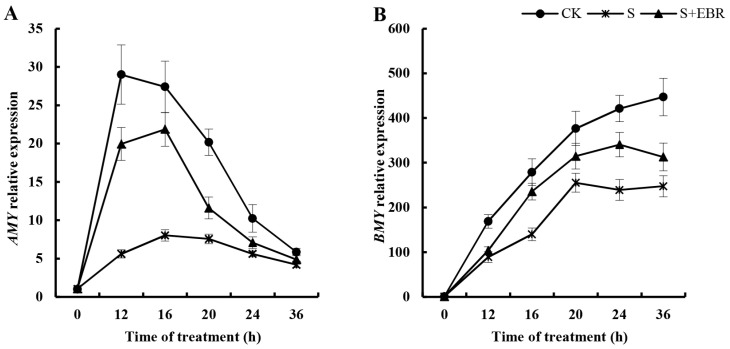
Effects of exogenous EBR on gene expression of AMY (**A**) and BMY (**B**) in the process of cucumber seed germination under NaHCO_3_ stress.

Figure 7 presents the fluctuating expression pattern of the *AMY* (amylase) gene in cucumber seeds during germination, characterized by an initial surge followed by a subsequent decrease. In contrast, the expression of *BMY* (beta-amylase) genes consistently increased. Exposure to NaHCO_3_ suppressed the activity of both *AMY* and *BMY* genes, with this effect being most pronounced within the first 24 h of germination.

Upon the application of exogenous EBR, there was an observed increase in the expression levels of both *AMY* and *BMY* genes in cucumber seeds under NaHCO_3_ stress. While EBR did not completely negate the suppressive effect of NaHCO_3_ on these genes, it played a significant role in mitigating their reduced expression. The influence of EBR on the *AMY* gene was particularly notable during the first 24 h of germination, whereas its effect on *BMY* gene expression extended throughout the 36 h period of the experiment. This indicates that EBR, while not fully reversing the NaHCO_3_-induced inhibition, contributes to a partial recovery of the expression of these key genes of amylolytic under alkaline stress.

**Figure 8:**
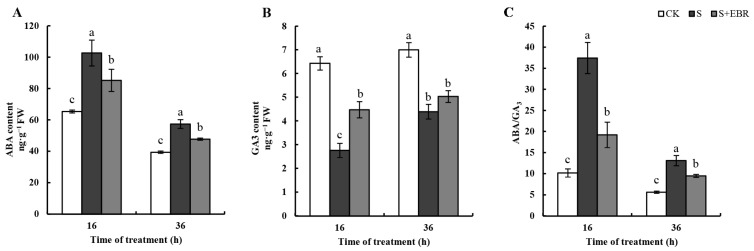
Effects of exogenous EBR on the content of ABA (**A**), GA3 (**B**), and ABA/GA3 ratio (**C**) in the process of cucumber seed germination under NaHCO_3_ stress. Different letters above bars indicate significance among different treatments as determined using Duncan’s samples comparison test (*p* < 0.05).

Figure 8 demonstrates that NaHCO_3_ application reduced the accumulation of ABA and GA3 in cucumber seeds while simultaneously increasing the ABA/GA3 ratio. Conversely, the application of external EBR decreased ABA levels and the ABA/GA3 ratio in stressed seeds, concurrently elevating GA3 levels.

**Figure 9:**
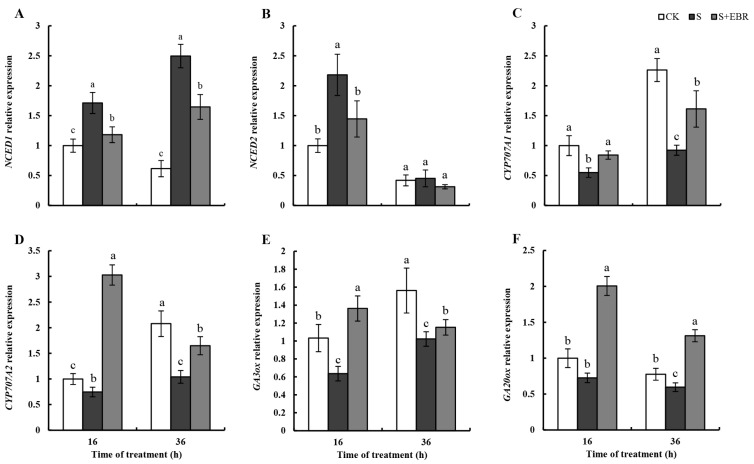
Effects of EBR on transcript levels of key genes involved in ABA and GA biosynthesis and catabolism in germinated cucumber seeds under NaHCO_3_ stress. The expression of ABA synthesis-related genes *NCED1* (**A**) and *NCED2* (**B**), GA biosynthesis-related genes *GA20ox* (**C**) and *GA3ox* (**D**), and ABA catabolism-related genes *CYP707A1* (**E**) and *CYP707A2* (**F**). Data are means ± SD with three biological replicate samples. Different letters above bars indicate significance among different treatments as determined using Duncan’s samples comparison test (*p* < 0.05).

Figure 9 reveals that NaHCO_3_ treatment upregulated the expression of ABA synthesis-related genes *NCED1* and *NCED2* and downregulated the ABA catabolism gene *CYP707A1* (*p* < 0.05), with minimal impact on *CYP707A2*. The application of exogenous EBR notably decreased the expression of *NCED1* and *NCED2*, indicating its role in suppressing ABA production and promoting ABA breakdown under NaHCO_3_ stress. This modulation in gene expression is instrumental in reducing ABA synthesis and enhancing its degradation, thereby reducing the suppressive impact of ABA during seed germination. Moreover, alkali stress suppressed the expression of *GA3ox* and *GA20ox* genes in cucumber seeds. However, the addition of exogenous EBR stimulated the expression of *GA3ox* and *GA20ox* genes, suggesting that EBR enhances the production of active GA via catalytic synthesis under alkali stress.

## 3. Discussion

Brassinosteroids (BRs), characterized as polyhydroxylated steroidal phytohormones, have gained recognition for their pivotal roles in modulating plant developmental trajectories, encompassing stages from flowering to germination, and extending their influence to optimize crop yields. Similarly, BRs, owing to their multifaceted attributes, occupy a central position in plant hormonal regulation. The adaptive machinery of plants is frequently engaged when confronted with an array of environmental stressors: extreme temperature deviations, elevated salinity, water scarcity, mechanical injury, fungal assaults, and challenges associated with metal toxicity. Such adversities instigate a cascade of phytohormonal responses, enabling plants to negotiate both abiotic and biotic impediments. Significant results of these stressors are the intensified production of ROS, encompassing superoxide ions and peroxides. Such unchecked ROS can jeopardize cellular integrity and functionality. Strategically timed exogenous applications of BRs can augment the inherent defense mechanisms of plants, offering them a bulwark against the oxidative repercussions of stress. In the face of environmentally inimical conditions, BRs fortify the plant’s capabilities by amplifying carbon dioxide assimilation, invigorating chlorophyll synthesis, and accentuating the reserves of key antioxidants like ascorbic acid, carotenoids, and proline. Transcending their role as mere stress mitigators, BRs also orchestrate vital growth processes, thereby catalyzing seed germination and ensuring the timely maturation of fruits [27].

Seed vigor and the germination rate stand as paramount indices when evaluating the germinative attributes of seeds, capturing nuances of both the tempo and homogeneity of germination, complemented by the latent vigor of ensuing seedlings [28]. Existing studies clearly demonstrate that the impact of alkaline salt stress surpasses that of neutral salts in hindering the germination processes of oilseed rape (*Brassica napus* L.) and *Chenopodium glaucum* [28,29]. Amplifying this, elevated salt concentrations discernibly attenuate the germination characteristics and vegetative propagation of hemp (*Cannabis sativa*), with the repercussions being more pronounced from alkaline salts compared to neutral variants [30]. In congruence with these insights, our present research documented a curtailment in cucumber seed germination in tandem with augmented alkali concentrations (Figure 1). This observation resonates with antecedent studies spotlighting the inverse dynamics between Cannabis sativa seed germination and escalating alkali salinity.

Recent studies highlight a notable increase in maize (*Zea mays* L.) germination under salt-induced stress, particularly following BR seed pretreatment [31]. Our research supports these observations. We demonstrate that exogenous EBR effectively mitigates the negative effects of NaHCO_3_ stress on cucumber seed germination. The resultant amplified germination rates and vigor of seeds parallel earlier investigations on BRs modulatory effects in rice (*Oryza sativa* L.) [32] as well as maize (*Zea mays* L.) [31]. Furthermore, these compounds have been identified to counter salt and drought stress in both barley (*Hordeum vulgare* L.) [32] and cucumber (*Cucumis sativus* L.) [33] samples. Complementing this, studies have pinpointed the role of BRs in attenuating seed germination setbacks instigated by salt stress, suggesting a potential interaction with the ethylene synthesis pathway [33]. 

According to reports, BR supplementation counteracts the negative consequences of water stress during the germination process of radish (*Raphanus sativus*) seeds [34]. Harmonizing with outcomes from salt-stressed quinoa (*Chenopodium quinoa*) seeds [35]. Within our experimental confines, the repercussions of NaHCO_3_ stress were evident in the slowed germination rates and curtailed seed hydration, manifesting as reduced fresh weights relative to controls. However, the integration of exogenous EBR effectively countered these effects, enhancing cucumber seed weight and fostering embryonic axis elongation, as illustrated in Figure 2. Such transformations likely originate from BRs’ regulatory interplay with cellular division and *MDP40* phosphorylation, influencing microtubule configurations [36].

Salinity stress disrupts cell membrane function and triggers an upsurge in ROS accumulation [1]. Our study revealed that exposure to NaHCO_3_ stress provoked a marked increase in ROS, resulting in oxidative damage accompanied by a spike in MDA concentrations within cucumber seeds. Impressively, the introduction of exogenous EBR counteracted these effects, markedly reducing ROS and MDA levels. This suggests that EBR not only alleviates the oxidative damages induced by alkali stress but also reinforces the cell membrane’s stability. Advanced plants employ sophisticated antioxidant systems as defense mechanisms against oxidative threats [37]. Central to these systems are vital antioxidant molecules, such as AsA and GSH, reinforced by the collective actions of enzymes like SOD, POD, and CAT, known for their antioxidant properties [38]. Previous literature has highlighted EBR’s capacity to modulate these antioxidant systems, efficiently neutralizing excessive reactive oxygen species during stress and thereby bolstering plant resilience [39,40,41,42]. Echoing these findings, our experiments demonstrated that EBR application under NaHCO_3_ stress conditions amplified the key antioxidant enzyme activities of cucumber seeds while concomitantly suppressing ROS and MDA levels. Germination relies heavily on amylases that metabolize stored starch in plant seeds into simpler sugars, which then fuel vital physiological processes [14,43,44]. This enzymatic conversion of starch is crucial for effective seed germination [44]. Amylase, ubiquitous in plants, efficiently hydrolyzes starch by cleaving its glycosidic linkages, yielding simpler sugars [45,46]. Its activity peaks in germinating seeds where α-amylase predominates, spearheading starch decomposition. This enzymatic intervention releases reducing sugars, primarily maltose and dextrin. Subsequently, α-amylase further processes maltose and catalyzes dextrin saccharification. It is noteworthy that stressful environmental factors can suppress amylase activity in germinating cucumber seeds [47]. Contrastingly, exogenous BRs have been documented to invigorate α-amylase activity even amidst such adversities [31,48,49]. BRs’ seed priming significantly enhanced maize (*Zea mays* L.) germination metrics and α-amylase activity in salt-stressed soil, increasing cumulative germination; conversely, it reduced germination time, days to 50% emergence [32]. In our investigations, we discerned that NaHCO_3_-induced stress diminished amylase functionality in cucumber seeds. This hindered the starch conversion in cotyledons, jeopardizing normal germination. Intriguingly, when augmented with exogenous EBR, the seeds exhibited rejuvenated amylase activity despite the NaHCO_3_ stress, fostering starch metabolism during germination. This observed resurgence could be ascribed to EBR’s potency in bolstering the antioxidant defenses of cucumber seeds, mitigating oxidative perturbations, and fostering an amenable physiological ambiance for amylase.

Abscisic acid (ABA) and gibberellic acid (GA) are fundamental hormones directing seed germination. Their dynamic interplay is pivotal in the germination continuum, with the equilibrium between their synthesis and degradation being especially influential [50,51,52]. As seeds initiate germination, ABA concentrations decline while GA levels ascend, leading to the cessation of dormancy and the commencement of germination [50,52,53]. Notably, alkaline stress can obstruct ABA degradation, thereby thwarting germination. The recent literature underscores the capacity of brassinosteroids (BRs) to counterbalance the repressive effects of ABA on germination. Furthermore, an intact BR signaling pathway appears indispensable for negating ABA-mediated germination restraint [12]. In our study, the application of 24-epibrassinolide (EBR) resulted in the downregulation of ABA synthesis genes, namely *NCED1* and *NCED2*, but simultaneously upregulated the transcription of genes central to ABA breakdown, *CYP707A1* and *CYP707A2*. This dual mechanism curbed ABA synthesis, accelerated its breakdown, and thus attenuated its overall concentration, providing a respite from the germination constraints of alkaline stress. It is worth noting that EBR’s modulation of the *CYP707A2* gene—crucial for ABA metabolism—was particularly pronounced [54,55]. In the realm of plant developmental biology, BRs and GA share overlapping regulatory functions. Genetic mutants with disrupted BR or GA pathways or heightened sensitivity thereto consistently manifest a suite of characteristics, including compromised germination, stunted growth, and deferred flowering [12,56,57]. In our experiments, we discerned that EBR heightened the transcriptional activity of primary GA biosynthetic genes *GA20ox* and *GA3ox* under alkaline duress. This action bolstered GA synthesis in cucumber seeds, fortifying their germinative potential. Given that BR administration is known to induce an upsurge in hydrogen peroxide (H_2_O_2_) concentrations and considering H_2_O_2_’s role in facilitating ABA metabolism and GA synthesis [58], this might offer a mechanistic insight into EBR’s germination-enhancing properties under alkaline conditions.

## 4. Materials and Methods

### 4.1. Experimental Materials and Design

This study employed “JinYan 4” (*Cucumis sativus* L.) as the cucumber variety for experimentation. The experimental trials were conducted at Shandong Agricultural University, Tai’an, China. 24-Epibrassinolide (EBR) was procured from Sigma-Aldrich, Burlington, VT, USA.

Adopting the approach by Božena ŠERÁ [59], our study involved a specific pretreatment of cucumber seeds. These seeds were first immersed in solutions with varying concentrations of EBR (24-epibrassinolide), as well as a control group in distilled water, maintaining a consistent temperature of 28 °C for 12 h. After immersion, the seeds underwent a sterilization process, exposed to a 0.5% (*v*/*v*) sodium hypochlorite solution for 10 min. This was followed by a series of five extensive rinses with distilled water to eliminate any residual sterilizing agent. Subsequently, the seeds were carefully placed in Petri dishes, each lined with three layers of filter paper to promote uniform conditions for germination. We introduced 10 mL of the respective treatment solution into each dish, ensuring a consistently moist environment for the duration of the experiment. The experimental design included three replicates for each treatment, with each replicate comprising 200 seeds. Germination was carried out in a dark environment at a stable temperature of 28 °C. Critical measures such as germination rates and various physiological indices were systematically recorded at set intervals after the treatment, aiming to evaluate the influence of different EBR concentrations on seed germination.

To assess the impacts of varying degrees of alkali stress, a comprehensive screening experiment was designed involving seven distinct concentrations of sodium bicarbonate (NaHCO_3_). The groups subjected to treatment included (1) 0 mmol L^−1^ NaHCO_3_ (control with distilled water), (2) 25 mmol L^−1^ NaHCO_3_, (3) 50 mmol L^−1^ NaHCO_3_, (4) 75 mmol L^−1^ NaHCO_3_, (5) 100 mmol L^−1^ NaHCO_3_, (6) 150 mmol L^−1^ NaHCO_3_, and (7) 200 mmol L^−1^ NaHCO_3_.

In subsequent experiments, we assessed the efficacy of exogenous 2,4-epibrassinolide (EBR) in alleviating the repressive influence of alkaline stress on the germination of cucumber seeds. This experiment encompassed three distinct treatments: (1) CK (control, distilled water), (2) S (75 mmol L^−1^ NaHCO_3_), and (3) S + EBR (0.2 μmol L^−1^ EBR with 75 mmol L^−1^ NaHCO_3_). Seed pretreatment involved immersion in either distilled water or a 0.2 μmol L^−1^ EBR solution for 12 h. 

### 4.2. Determination of Seed Germination Rate and Fresh Weight

To accurately assess the efficacy of the different treatments, the seed germination rate was systematically documented at predefined intervals post-treatment, specifically at 0, 12, 16, 20, 24, and 36 h. In this analysis, the emergence of the radicle from the seed coat was employed as the definitive criterion for germination. This methodology facilitated an in-depth examination of both the germination timeline and the corresponding responses to the treatments. Additionally, the fresh weight of the seeds, which had successfully germinated, was quantitatively measured at the 36 h mark only.

For the calculation of germination percentages, we adhered to the method proposed by Božena ŠERÁ [59]: Germination (%) = [100 × (number of germinated seeds/total number of seeds)]. This approach provided a standardized measure for comparing germination across different treatments.

### 4.3. Assessment of Seed Viability

The viability of the seeds was ascertained by employing the 2,3,5-triphenyltetrazolium chloride (TTC) staining technique, a technique well documented in previous research [60,61]. After a 36 h treatment, the seeds were carefully extracted and bisected along the embryonic axis. The radicle was removed, and the halves were then submerged in a 0.1% (*w*/*v*) TTC solution. Subsequently, the seeds underwent a one-hour incubation period at ambient temperature. Post-incubation, a series of three thorough water rinses were applied to the seeds. The staining results were visually evaluated and documented photographically. Each treatment was replicated thrice, with each replicate comprising 20 germinated seeds.

### 4.4. Determination of Reactive Oxygen Species (ROS) Levels and MDA Contents

The O_2_^·−^ production rate was determined following Xia’s method [62]. Fresh seeds (1 g) underwent homogenization in a phosphate-buffered solution (pH 7.8), followed by centrifugation at 4000× *g* for a quarter-hour, subsequently reacting with hydroxylamine. After incubation, α-naphthylamine solution and p-aminobenzene sulfonic acid were added to the reaction mixture, followed by further incubation. The measurement of absorbance at 530 nm facilitated the calculation of the quantification of H_2_O_2_ levels in a NaNO_2_ standard curve. The quantification of H_2_O_2_ levels was assessed using Gong’s method [4], which involved the quantification of absorbance for the titanium peroxide complex at 415 nm.

The content of malondialdehyde (MDA) is indicative of lipid peroxidation measured according to the protocol of Hodges et al. [63]. Fresh seed tissue (1 g) was homogenized in a 10% (*v*/*v*) trichloroacetic acid (TCA) solution. Subsequently, the homogenate underwent centrifugation at 4000× *g* for a duration of 10 min. Following this, the supernatant was combined with 0.6% thiobarbituric acid (TBA) and heated in a boiling water bath for 15 min. Following cooling, it was centrifuged again. The absorbance levels of the resulting supernatant were quantified at 532 nm, 600 nm, and 450 nm for the assessment of MDA content, subsequently calculated using a standard linear equation.

### 4.5. Determination of Antioxidant Enzyme Activity

The quantification of antioxidant enzyme SOD, POD, and CAT activities was conducted following the protocol described by Shalata et al. [64]. Fresh samples of germinating cucumber seeds weighing 0.3 g were homogenized in 3 mL of 50 mM phosphate-buffered saline (PBS) buffer (pH 7.8) containing 2 mM ascorbate, 0.2 mM EDTA, and 2% polyvinylpyrrolidone. The homogenate was then centrifuged at 4 °C and 12,000× *g* for 20 min, with the supernatant used for enzyme activity assays. SOD activity was indicative of its capacity to obstruct the photochemical reduction of nitroblue tetrazolium (NBT). The measurement was taken at 560 nm following the protocol described by Gong et al. [4]. In this context, a 50% decrease in NBT photoreduction was considered equivalent to one unit of enzymatic activity”. The activity of POD was ascertained by recording the increase in absorbance at 470 nm, indicative of guaiacol oxidation, following Shalata et al.’s methodology [64]. CAT activity was quantified by monitoring the reduction in absorbance noted at 240 nm, correlating with the degradation of hydrogen peroxide (H_2_O_2_), according to the approach of Shalata et al. [64].

### 4.6. Determination of Abscisic Acid (ABA) and Gibberellin (GA_3_) Content

Following the methodology outlined by Chen and colleagues [65], we accurately measured the levels of ABA and GA3 at different stages of germination in cucumber seeds using ELISA (enzyme-linked immunosorbent assay). Utilizing mlbio’s Plant ABA ELISA KIT and Plant GA ELISA KIT, hormone levels were assessed at critical post-treatment intervals of 16 and 36 h. This was carried out across three independent biological replicates to ensure the reliability of the data.

Initially, each batch of cucumber seeds, weighing 0.3 g, was swiftly frozen using liquid nitrogen, followed by blending with 2 mL of the extraction buffer supplied in the kit. The samples, kept in an ice-cold environment, were homogenized and subsequently transferred into 10 mL centrifuge tubes. For thorough sample transfer, the mortar received an extra 2 mL of extraction solution rinse, with these washings then being amalgamated with the tube-contained homogenate, succeeded by intense shaking. The mixture was chilled at 4 °C for a duration of 4 h to allow phase separation and subsequently subjected to centrifugation at a force of 1000× *g* for a quarter of an hour. The upper liquid layer was then cautiously extracted, and a volume of 1 mL was fused back with the sediment, agitated rigorously, and exposed to additional extraction at a temperature of 4 °C across 60 min. After another round of centrifugation for 15 min, the combined supernatant was measured for its total volume. Purification of the extract was conducted utilizing a cartridge of Agilent Bond Elut C18 for Solid Phase Extraction (SPE). Subsequently, the purified eluent was relocated into a centrifuge tube of 5 mL and concentrated by applying a vacuum or inducing evaporation of methanol via nitrogen gas. Once concentrated, the sample was diluted to a specified volume according to the kit’s instructions. For the final assay, the prepared samples were incubated with specific antibodies, and absorbance was quantitatively determined at 492 nm using an enzyme marker (Thermo Multiskan FC, Thermo Fisher Scientific, Waltham, MA, USA). This comprehensive procedure enabled the accurate measurement of ABA and GA concentrations.

### 4.7. Determination of Amylase Activity and Starch Content

Determination of Starch content: In accordance with the esteemed methodology delineated by Allfrey and Northcote [66], the quantification of starch content was meticulously executed. The experimental protocol commenced with the meticulous homogenization of 36 cotyledon pairs or axes in a 16 mL aliquot of 80% (*v*/*v*) ethanol, employing an ice-cold mortar and pestle to ensure optimal conditions. This was followed by a centrifugation step, exerting a force of 30,000× *g* for 10 min at a controlled temperature of 2 °C to effectively separate the components. Subsequent to this, 6 mL of 30% (*v*/*v*) perchloric acid (HClO_4_) was judiciously added to the homogenate, facilitating the dissolution of starch within the granules. The solution was then allowed to equilibrate at ambient temperature for a duration of 6 h, setting the stage for the starch assay. For the quantification, a bespoke I2-KI reagent was prepared, entailing the dilution of 0.1 mL of a concentrated stock solution (comprising 0.06 g I2 and 0.60 g KI in 10 mL deionized water) with 0.05 M HCl, tailored specifically for this analysis. A volume of 0.5 mL from the starch solution was meticulously mixed with an equal volume of the I2-KI reagent, followed by the addition of 1 mL of 30% (*v*/*v*) perchloric acid. The resultant mixture was vortexed and allowed to stabilize at room temperature. The critical assessment of starch concentration was conducted by measuring the absorbance at 620 nm, correlating it with a meticulously established standard curve ranging from 0 to 5 mg/mL, using the identical I2-KI reagent derived from starch solubilized in 30% HClO_4_. Rigor was maintained by repeating the analysis for each sample in triplicate, ensuring the reliability and precision of the data obtained.

Determination of Amylase activity: The extraction of total amylase followed the method outlined by Li and colleagues [67]. In conditions of 4 °C, 0.3 g of freshly germinated cucumber seed samples underwent homogenization in 2 mL of acetate buffer (0.05 M, pH 6.0) utilizing a pestle. The homogenate obtained was first passed through cheesecloth, subsequently centrifuged at 18,000× *g* for 10 min, and then subjected to filtration. The protocol for α-amylase preparation was largely akin to that for total amylase extraction, except it involved an additional step of heating at 70 ± 0.5 °C for a duration of 15 min subsequent to filtration. For conducting amylase assays, the employed substrate comprised a 1% soluble starch solution in 0.1 mol L^−1^ acetate buffer, maintaining a pH of 5.6. The enzyme solution prepared was initially diluted to a volume of 1 mL using water, which was then supplemented with 1 mL of starch solution. This mixture was incubated at a constant temperature of 25 °C for one hour. Following incubation, 2 mL of 3,5-dinitrosalicylic acid reagent was introduced into the mixture. The tubes containing this reaction mixture were then heated in a boiling water bath for 5 min. Post-heating, the tubes were allowed to return to room temperature and were further diluted with 20 mL of water. The intensity of the colored solutions was quantified using a spectrophotometer. The intensity of the colored solutions was quantified using a spectrophotometer. β-amylase was calculated from the difference between α-amylase and the total contents of amylase.

### 4.8. Gene Expression Analysis

The isolation of total RNA from these seeds using an RNA extraction kit from Invitrogen using the TRIzol method. Assessments of RNA quality and quantity were carried out via 1% agarose gel electrophoresis, complemented by measurements using a NanoDrop Photometer spectrophotometer (IM-PLEN, Westlake Village, CA, USA). This process preceded the reverse transcription of the isolated RNA into cDNA, utilizing the Super-Script™ First-Strand Synthesis System for RT-PCR kit (18091050; Invitrogen, Waltham, MA, USA). Subsequently, the cDNA underwent qRT-PCR (quantitative real-time PCR), with primer sequences meticulously designed as delineated in Table 1. Actin served as an internal control. Primer designs for qRT-PCR were facilitated using Primer Premier 5 software (Biosoft International, Palo Alto, CA, USA). The qRT-PCR experiments utilized the ABI Prism 7900 HT system (Applied Biosystems, Waltham, MA, USA). The ascertainment of relative gene expression levels was achieved via the 2^−ΔΔCT^ method, with analysis conducted using the 7500 software v2.0.6 (Applied Biosystems).

### 4.9. Data Processing

In this research, a fully randomized experimental design was implemented, with each treatment group undergoing three sets of replications. We represented the data as mean values accompanied by the standard error. For identifying significant variances among the means, Duncan’s New Multiple Range Test, as facilitated by SPSS software version 22.0, was employed. Statistical significance was inferred when the *p*-value was lower than 0.05. Additionally, for further data analysis and chart creation, Excel 2010 was utilized.

## 5. Conclusions

**Figure 10:**
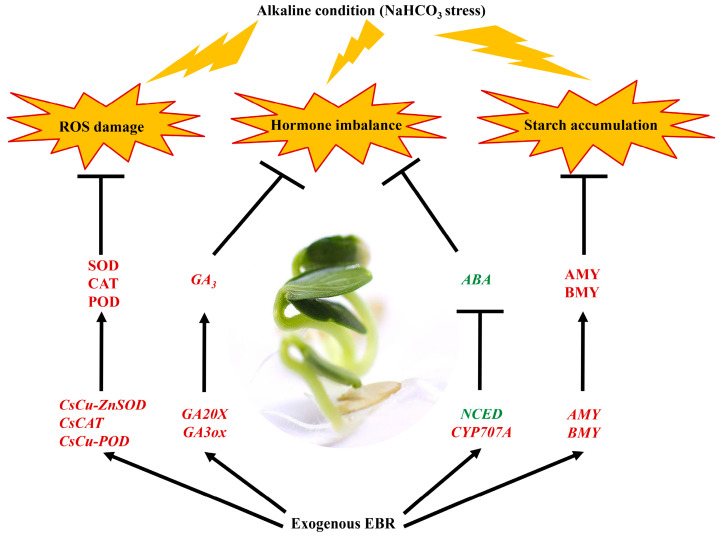
Schematic representation of three mechanisms by which exogenous EBR mitigates the inhibitory effects of NaHCO_3_ stress on the germination of cucumber seeds. EBR application results in ROS regulation, hormone balance, and starch accumulation. Genes or molecules highlighted in red indicate upregulation, while those in green signify downregulation in response to exogenous EBR.

The application of exogenous EBR effectively countered the negative impact of alkaline stress on cucumber seed germination. This positive outcome is attributed to EBR’s regulatory influence on the antioxidant defense system, its modulation of phytohormonal dynamics, and its governance over amylase activity. Given the multifaceted physiological process of seed germination, it is imperative to delve deeper into the nuanced regulatory roles of EBR, especially in the context of alkaline stress on cucumber seed germination (Figure 10).

## Figures and Tables

**Table 1:** Primers sequences.

Gene Name	Primer Sequences
*Actin*	F: CCCCGATGGGCAGGTAATA; R: AAGAGCAGGACGAACAGCAGA
*AMF*	F: CACGGTTATTACACCCAGGACT; R: TAAATCACTTGGTTGCCCAT
*BMF*	F: GGTGTCAAGTGGTAGCAACAATAAC; R: TGTCCTCTCTTTCTCTTCTAATGGTCT
*Cu/Zn SOD*	F: CAAGTTAACGCATGGTGCTC; R: GGCAGTTATGTTTCCCAGGT
*POD*	F: CAGGAAGGAGGGATGGTTT; R: TGGTGTTAGGTTCACTGTTGGA
*CAT*	F: ATGCTGGAAGAGGAGGCTAT; R: ATGGTGAGGACATTTGGGAG
*NCED1*	F: CAGGGGGTTATTTGGTCTTGTT; R: ATCATCGTTGGCTGAGGCA
*NCED2*	F: CAAATCCGAAGTTTAGCCCAG; R: CATAATCCAGCAGACCAAGCG
*CYP707A1*	F: TCGGAGTTCTGTTTGCGGCT; R: TGGTAAAGGGCATAGTTCGT
*CYP707A2*	F: CCCAACATCCAACCTCCT; R: CTCGGGCGTCGCTAACAT
*GA20ox*	F: ATCCGTTCCTTATGTTGCTG; R: CCTCATTATTGATTCATTGTCC
*GA3ox*	F: ATTCCCTCTTCTCCCTTCCT; R: ACGCAACCCACATCAGCC

## Data Availability

The study’s data can be obtained by contacting the corresponding author upon request.
